# Full-length optic nerve regeneration in the absence of genetic manipulations

**DOI:** 10.1172/jci.insight.164579

**Published:** 2023-04-10

**Authors:** Qian Feng, Kimberly A. Wong, Larry I. Benowitz

**Affiliations:** 1 Department of Neurosurgery, Boston Children’s Hospital, Boston, Massachusetts, USA.; 2 Department of Neurosurgery, Harvard Medical School, Boston, Massachusetts, USA.; 3 F.M. Kirby Neurobiology Center, Boston Children’s Hospital, Boston, Massachusetts, USA.; 4 Department of Ophthalmology, Harvard Medical School, Boston, Massachusetts, USA.

**Keywords:** Inflammation, Neuroscience, Monocytes, Neurological disorders

## Abstract

The inability of mature retinal ganglion cells (RGCs) to regenerate axons after optic nerve injury can be partially reversed by manipulating cell-autonomous and/or -nonautonomous factors. Although manipulations of cell-nonautonomous factors could have higher translational potential than genetic manipulations of RGCs, they have generally produced lower levels of optic nerve regeneration. Here, we report that preconditioning resulting from mild lens injury (conditioning LI, cLI) before optic nerve damage induced far greater regeneration than LI after nerve injury or the pro-inflammatory agent zymosan given either before or after nerve damage. Unlike zymosan-induced regeneration, cLI was unaltered by depleting mature neutrophils or T cells or blocking receptors for known inflammation-derived growth factors (oncomodulin, stromal cell–derived factor 1, CCL5) and was only partly diminished by suppressing CCR2^+^ monocyte recruitment. Repeated episodes of LI led to full-length optic nerve regeneration, and pharmacological removal of local resident macrophages with the colony stimulating factor 1 receptor inhibitor PLX5622 enabled some axons to reinnervate the brain in just 6 weeks, comparable to the results obtained with the most effective genetic manipulations of RGCs. Thus, cell-nonautonomous interventions can induce high levels of optic nerve regeneration, paving the way to uncovering potent, translatable therapeutic targets for CNS repair.

## Graphical abstract

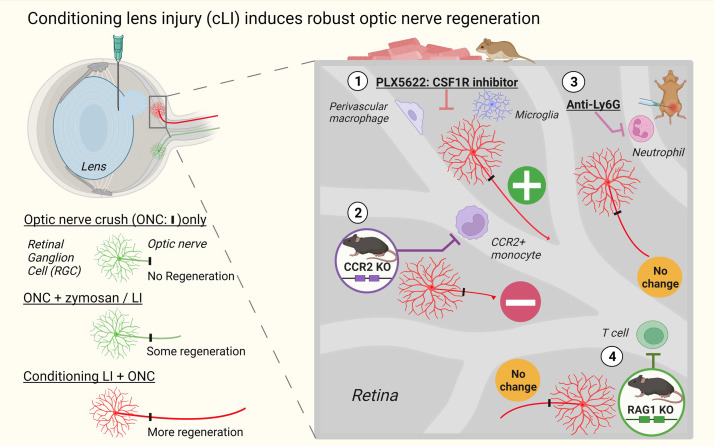

## Introduction

In adult mammals, retinal ganglion cells (RGCs), the projection neurons of the eye, cannot regenerate their axons after optic nerve injury and soon begin to die, resulting in permanent vision loss (1). Considerable effort has been directed toward identifying RGC-autonomous factors that suppress or enable optic nerve regeneration through candidate testing and in vivo multi-omics screening (1–5). Manipulating neuro-immune interactions for neuroprotection and enhancing axonal regeneration represent a new frontier for identifying potential therapeutic targets in many neurological diseases (6, 7).

Studies from our lab and others have shown that in rodents, ocular inflammation by lens injury (LI), intravitreal injection of zymosan, agonists of particular myeloid cell receptors, and a recently discovered population of immature neutrophils (but not LPS) partially protect RGCs from dying in the rodent optic nerve crush (ONC) model and stimulate axon regeneration through the action of oncomodulin (Ocm), stromal cell–derived factor 1 (SDF1), CCL5, and other factors immune cells express (8–16). However, long-distance regeneration and brain reinnervation are yet to be achieved by cell-nonautonomous manipulations alone, arguing for the urgent need to uncover more effective interventions.

Unlike RGCs, sensory neurons of the dorsal root ganglia can regenerate their peripheral axon branches after sciatic nerve injury, and a conditioning peripheral nerve injury potentiates the ability of sensory neurons both to regenerate their peripheral axon branches after a second injury and to regenerate their central axon branches after spinal cord injury (17, 18). This phenomenon is partly driven by infiltrative immune cells (19–22) and represents one of the strongest paradigms for spinal cord regeneration (23). Here, we investigated whether inflammatory preconditioning by zymosan or LI would enable axon regeneration in the mouse ONC model and examined whether perturbation of major immune cell types and known immune cell–derived growth factors affect regeneration using pharmacological or genetic manipulations.

## Results

### Preconditioning by LI induces robust optic nerve regeneration.

**Figure 1:**
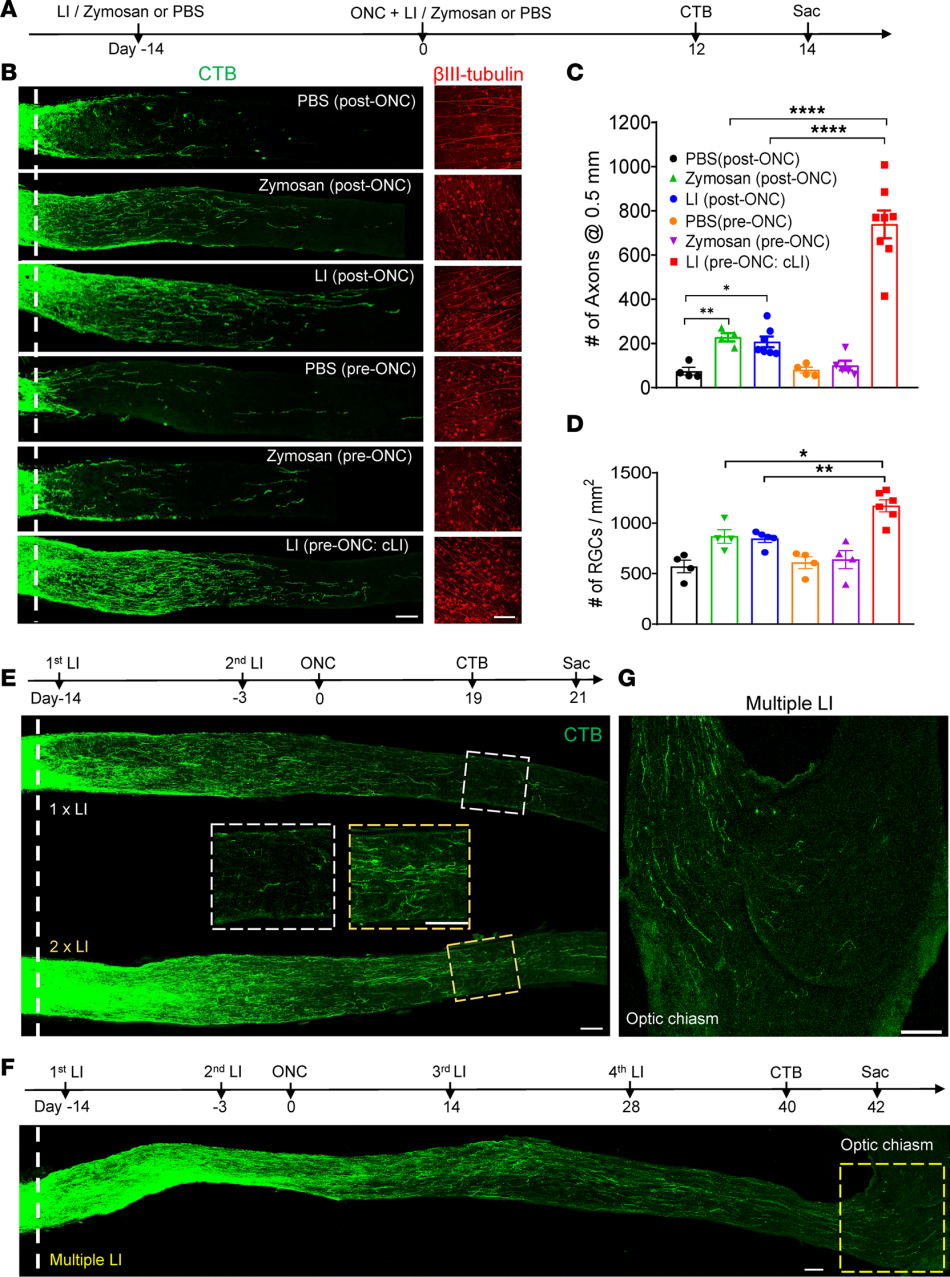
Robust optic nerve regeneration by conditioning LI. (**A**) Experimental timeline. LI, zymosan, or PBS was introduced 14 days before or immediately after optic nerve crush (ONC). (**B**) Left: representative longitudinal sections through the optic nerve showing CTB-labeled regenerating axons 14 days post-ONC. Preconditioning by LI before crush (LI pre-ONC: cLI) induced far greater regeneration than other treatments. Dashed white line, crush site. Right: whole-mounted retinas showing βIII-tubulin^+^ RGCs. Scale bar, 100 μm. (**C**) Quantitation of regenerating axons 0.5 mm from crush site in **B** by 1-way ANOVA followed by Tukey’s multiple comparisons test; PBS (post-ONC) vs. zymosan (post-ONC), *P* = 0.007; PBS (post-ONC) vs. LI (post-ONC), *P* = 0.0321; LI (post-ONC) vs. LI (pre-ONC), *P* < 0.0001; zymosan (post-ONC) vs. LI (pre-ONC), *P* < 0.0001, *n* = 4 or 5 mice in each group. (**D**) Quantitation of βIII-tubulin^+^ cells (surviving RGCs) in **B** by 1-way ANOVA followed by Tukey’s multiple comparisons test; LI (pre-ONC: cLI) vs. zymosan (post-ONC) *P* = 0.019; vs. LI (post-ONC) *P* = 0.0064; *n* = 4 or 5 mice in each group; 7–8 fields were analyzed for each retina. (**E**) Top: timeline: first LI 14 days before ONC, second LI 3 days before ONC, and 3-week survival after ONC. Bottom: optic nerve sections showing axon regeneration 3 weeks after ONC in mice treated with 1× versus 2× LI. Boxes in center: magnified images of axons 2 mm from crush site. White line, crush site. Both scale bars, 100 μm. (**F**) Top: experimental timeline as in **E** but with third and fourth LI 14 and 28 days post-ONC and a 6-week survival time after ONC. Bottom: sections showing full-length optic nerve regeneration. White line, crush site. Yellow dashed box, optic chiasm. Scale bar, 100 μm. (**G**) Enlarged image of optic chiasm. Scale bar, 100 μm. *P < 0.05, **P < 0.01, ****P < 0.0001.

Based on the role of inflammation in the peripheral nerve preconditioning phenomenon and our incidental observation that intraocular injections prior to ONC that inadvertently injure the lens result in strong regeneration, we sought to evaluate the pro-regenerative effects of LI or intraocular zymosan before versus after ONC (Figure 1A) in 129S1/SvImJ (129S1) mice. The controlled, mild LI used here damaged the lens capsule and cortex locally without causing global cataract formation (Supplemental Figure 1A; supplemental material available online with this article; https://doi.org/10.1172/jci.insight.164579DS1). As expected, zymosan or mild LI administered immediately after ONC (zymo post-ONC, LI post-ONC, respectively) led to moderate levels of optic nerve regeneration, whereas PBS or zymosan injected 2 weeks before ONC (PBS pre-ONC, zymo pre-ONC, respectively), had almost no effect (Figure 1, B and C). In contrast, conditioning LI 2 weeks before ONC (LI pre-ONC: cLI) nearly tripled the level of optic nerve regeneration compared with LI post-ONC or zymo post-ONC and increased RGC survival (Figure 1, B–D). Surprisingly, LI 1 week pre-ONC was less effective than at 2 weeks beforehand (Supplemental Figure 1B), yet 2 rounds of cLI at 14 days and again at 3 days before ONC (1× LI vs. 2× LI) doubled the number of regenerating axons compared with a single cLI (Figure 1E and Supplemental Figure 1C), pointing to the importance of timing and frequency of LI in regeneration. Therefore, we next evaluated the long-term effect of repeated episodes of LI by administering cLI 14 days and 3 days before ONC (first and second LI) and again at 14 and 28 days post-ONC (third and fourth LI). Multiple episodes of LI enabled many axons to regenerate the full length of the optic nerve and across the chiasm 6 weeks after ONC (Figure 1, F and G). Together, these results demonstrate that cLI induces robust optic nerve regeneration and that full-length optic nerve regeneration can be achieved with multiple LIs in the absence of genetic manipulations.

### Roles of major immune cell populations.

Previous studies have reported that microglia are irrelevant for optic nerve regeneration induced by LI post-ONC (24), that zymosan-induced regeneration is mediated primarily by infiltrative neutrophils and macrophages (10–12), and that T cells are neuroprotective in the ONC model (25). To understand neuroimmune interactions, we therefore investigated the effects of removing major immune cell populations on cLI-induced regeneration using pharmacological depletion or genetically altered mice.

### Removing naive resident macrophages with colony stimulating factor 1 receptor inhibitor PLX5622 further enhances regeneration.

**Figure 2:**
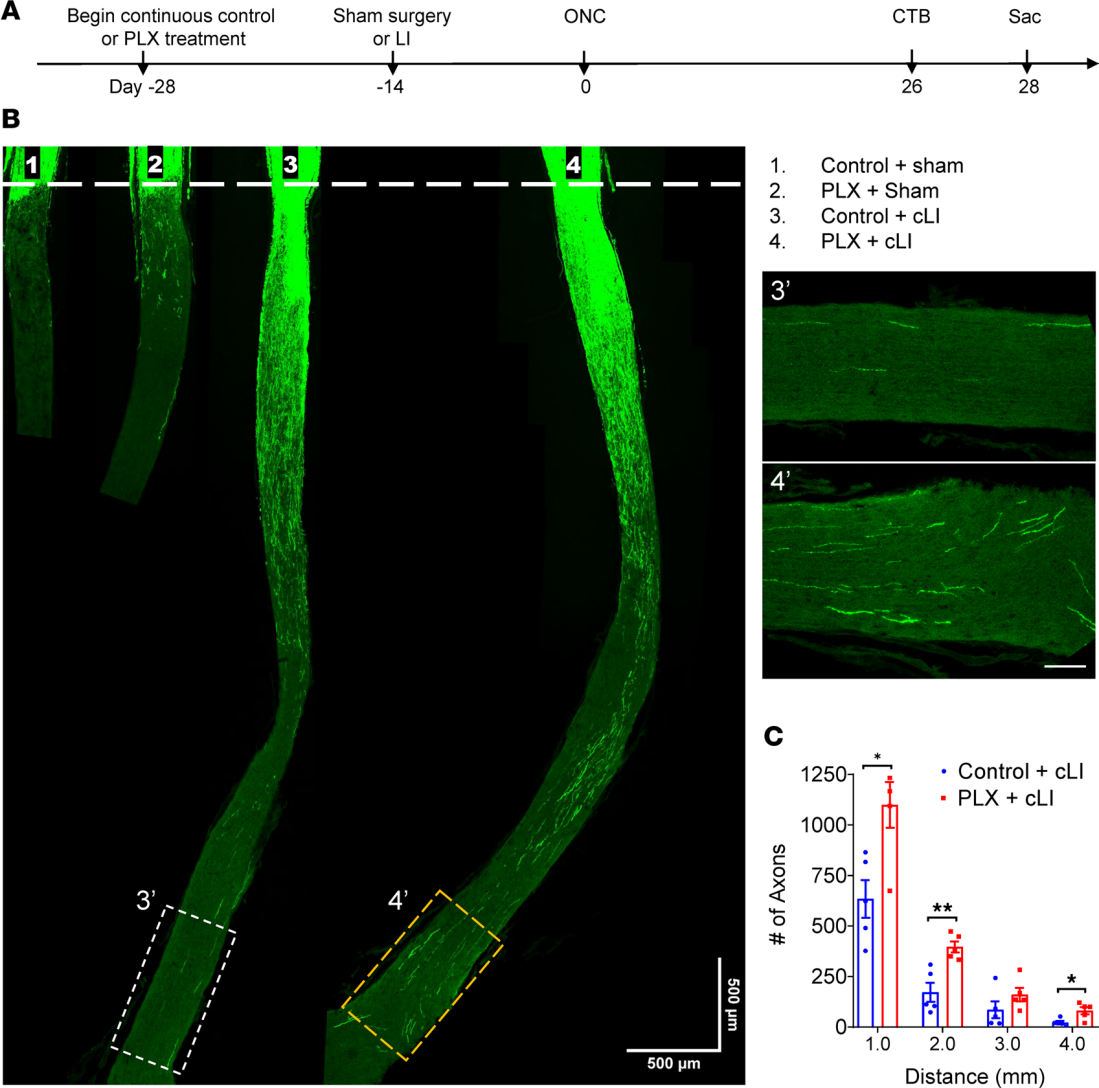
Removing naive resident macrophages with CSF1R inhibitor PLX5622 further enhances cLI-induced regeneration. (**A**) Experimental timeline. Mice were fed with PLX5622 (PLX) or control chow from 14 days prior to LI or sham surgery until time of euthanasia. LI or sham surgery was performed 14 days before ONC, and mice were euthanized 4 weeks later. CTB, cholera toxin B fragment. (**B**) Representative longitudinal sections through the optic nerve showing CTB-labeled regenerating axons day 28 post-ONC. Note increase in regeneration when cLI is combined with PLX treatment. White line indicates the crush sites. Scale bar, 500 μm. Insets at right show magnified images of regenerating axons near optic chiasm in white and yellow dashed boxes from third and fourth sections. Scale bar, 100 μm. (**C**) Quantitation of regenerating axons at multiple distances from crush site in **B** (unpaired *t* test, *P* = 0.0131 at 1 mm; 0.0035 at 2 mm, 0.200 at 3 mm, and 0.018 at 4 mm. *n* = 5 mice per group). *P < 0.05, **P < 0.01.

Resident CNS macrophages consist of several cell populations in distinct niches, including microglia, border-associated macrophages (meningeal macrophages), and perivascular macrophages. Targeting specific populations remains challenging due to shared markers like ionized calcium-binding adaptor molecule 1 (Iba1) or CD68 and receptors like colony stimulating factor 1 receptor (CSF1R). To deplete resident macrophages, mice were fed with chow containing the CSF1R inhibitor PLX5622 starting 4 weeks prior to ONC (i.e., 2 weeks before cLI or sham surgery), whereas control mice were fed chow without the drug (Figure 2A) continuously until sacrifice. In mice fed control chow, cLI doubled the number of Iba1^+^ cells in the retina compared with sham surgery controls. PLX nearly eliminated these cells throughout the retina after ONC and sham surgery, though after cLI, a small population of Iba1^+^ cells remained (Supplemental Figure 2, A and C). In the optic nerve, on the other hand, colabeling with anti-CTB and -CD68 antibodies revealed a population of CD68^+^ cells persisting with PLX treatment after either sham surgery or cLI, particularly around the crush site (Supplemental Figure 2D; note that these latter studies combined immunostaining for CTB with staining for CD68, a marker for phagocytic cells, because the available antibody for Iba1 was generated in the same species, rabbit, as the antibody for CTB). Although PLX treatment alone did not promote regeneration (Supplemental Figure 2G), PLX combined with cLI nearly doubled the number of axons extending the full length of the optic nerve (≥4 mm beyond the injury site) compared with cLI alone when examined 4 weeks after ONC (Figure 2, B and C), though with no effect on RGC survival (Supplemental Figure 2, A and B).

Despite being a highly selective brain-penetrant CSF1R inhibitor, PLX is reported to also affect peripheral myeloid cell populations in C57BL/6 mice (26). Here, we found that PLX treatment for 4 weeks in 129S1 mice increased the percentage of circulating monocytes (CD11b^+^Ly6C^+^Ly6G^lo^ cells) in blood, without affecting the overall myeloid cell population (CD11b^+^ cells: Supplemental Figure 2, E and F).

### Blocking CCR2^+^ monocytes partially decreases the preconditioning effect.

**Figure 3:**
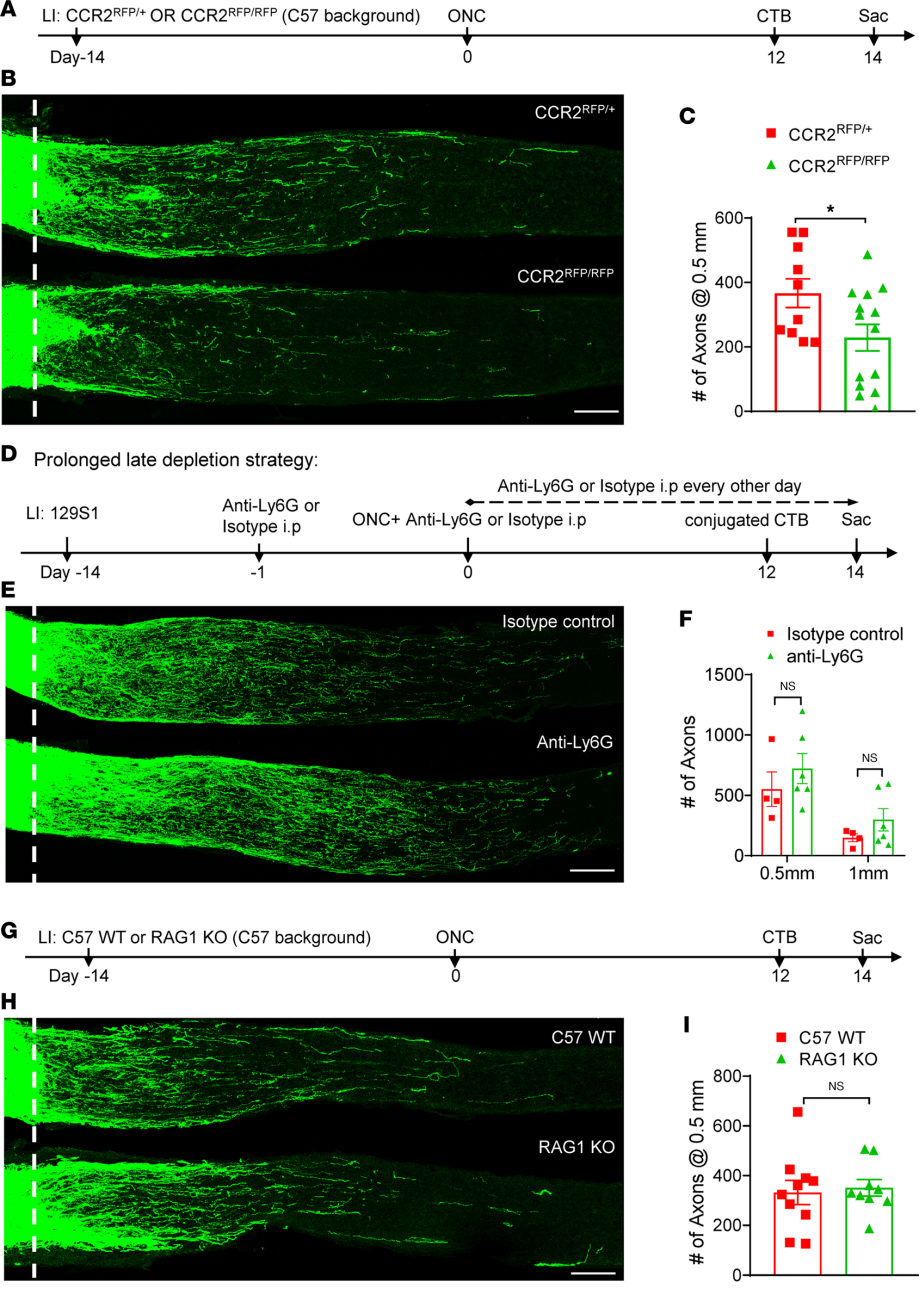
Blocking CCR2^+^ monocytes partially suppresses cLI-induced regeneration, whereas mature neutrophils and T cells are dispensable. (**A**) Experimental timeline. LI was introduced to CCR2^RFP/+^ or CCR2^RFP/RFP^ mice with C57BL/6 background 14 days before ONC. Mice were euthanized 14 days after ONC. (**B**) Representative longitudinal sections through the optic nerve showing decreased regeneration in CCR2^RFP/RFP^ versus CCR2^RFP/+^ mice. White line, crush site. Scale bar, 100 μm. (**C**) Quantitation of regenerating axons (unpaired *t* test, *P = 0.0357; *n* = 6 or 7 mice per group). (**D**) Experimental timeline for prolonged late depletion strategy. LI was introduced to 129S WT mice 14 days before ONC. Mice were euthanized 14 days after ONC. (**E**) Representative longitudinal sections through the optic nerve show similar levels of regenerating axons in isotype control and neutrophil-depleted mice. White line, crush site. Scale bar, 100 μm. (**F**) Quantitation of regenerating axons 0.5 mm from crush site in **E** (unpaired *t* test, *P* = 0.402 at 0.5 mm, *n* = 4 or 5 mice per group). (**G**) Experimental timeline. LI was introduced to C57BL/6J WT mice or RAG1-KO mice 14 days before ONC. Mice were euthanized 14 days after ONC. (**H**) Longitudinal sections through the optic nerve showing similar levels of regenerating axons in C57BL/6J WT and RAG1-KO mice. White line, crush site. Scale bar, 100 μm. (**I**) Quantitation of regenerating axons 0.5 mm from crush site in **H** (unpaired *t* test, *P* = 0.758, *n* = 5 or 6 mice per group).

To investigate the role of peripheral monocytes, we suppressed the infiltration of a subset of these cells using mice genetically deficient in CCR2, a CC chemokine receptor that is important for leukocyte egress from bone marrow and that is highly expressed in blood monocytes but absent in resident CNS macrophages (27). Mice with replacement of CCR2 by red fluorescent protein [B6.129(Cg)-CCR2tm2.1Ifc/J; CCR2^RFP/RFP^] with a C57BL/6 background were used as CCR2-deficient reporter mice. Following cLI and ONC, these mice showed an 84% reduction in circulating CCR2^+^ monocytes (RFP^+^ cells in blood) (27) and an 80% reduction in vitreous infiltrating CCR2^+^ monocytes (RFP^+^ cells in the vitreous chamber) compared with heterozygous CCR2^RFP/+^ littermate controls (Figure 3A and Supplemental Figure 3, A and B). CCR2^RFP/RFP^ mice showed a 38% decline in cLI-induced regenerating axons 2 weeks after ONC (Figure 3, B and C) compared with heterozygous CCR2^RFP/+^ controls without diminishing RGC survival (Supplemental Figure 3, C and D). It should be noted that, in this set of experiments, we found a lower baseline level of regeneration than in 129S1 mice, consistent with previously reported variations in regenerative capacity among different mouse strains (28, 29).

### Mature neutrophils and T cells are dispensable for the preconditioning effect.

In mice, mature neutrophils are identified as CD11b^+^Ly6G^hi^Ly6C^intermediate^ cells. To evaluate their contribution to the preconditioning effect, we used multiple strategies to deplete mature neutrophils using an anti-Ly6G antibody in 129S1 mice. Transient mature neutrophil depletion (Supplemental Figure 3E) was achieved by injecting mice twice retro-orbitally (3 days before and once after ONC) and twice i.p. (immediately after and 7 days after ONC) with the antibody. For prolonged early neutrophil depletion (Supplemental Figure 3F), mice were injected 1 day before and once after cLI and every other day thereafter until ONC. For prolonged late neutrophil depletion (Figure 3D), mice were injected with anti-Ly6G 1 day before and once after ONC and every other day thereafter until euthanasia, at which time blood was collected for flow analysis. Mature neutrophils were almost completely absent after immune depletion (Supplemental Figure 3, G and H), yet all 3 strategies failed to reduce the number of regenerating axons induced by cLI (prolonged late depletion: Figure 3, E and F), suggesting mature neutrophils are not required for the preconditioning effect.

Finally, we tested whether mature T cells contribute to the effects of cLI using recombination activating 1–knockout (B6.129S7-*Rag1^tm1Mom^*/J; RAG1-KO) mice that lack mature T cells because of these cells’ impaired development. RAG1-KO mice showed the expected loss of mature T cells by CD3 staining in the retina following cLI and ONC (Figure 3G and Supplemental Figure 3I). This did not, however, alter cLI-induced regeneration (Figure 3, H and I).

Taken together, these data demonstrate that removing naive resident macrophages with PLX5622 further enhances cLI-induced optic nerve regeneration, while blocking CCR2^+^ monocytes because of CCR2 deficiency partially decreases regeneration, and that mature neutrophils and T cells are unlikely to contribute substantially to cLI-induced regeneration. Manipulations to alter major immune populations are illustrated schematically later in the manuscript.

### cLI activates pro-regenerative signaling pathways in injured RGCs but does not rely on Ocm, SDF1, or CCL5.

**Figure 4:**
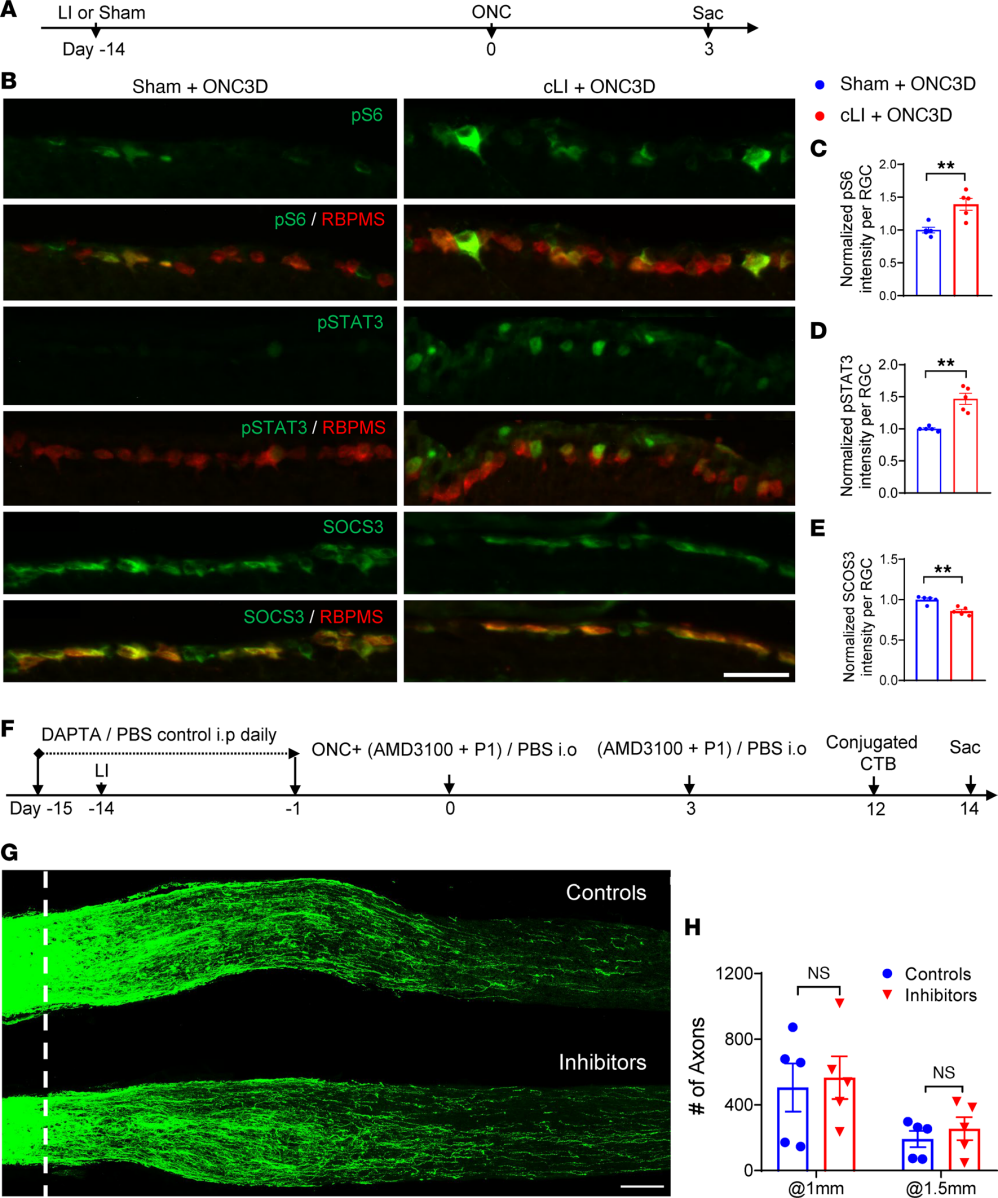
mTOR and STAT3 act as possible downstream signaling pathways for cLI-induced regeneration with minimal contributions of Ocm, SDF1, and CCL5. (**A**) Experimental timeline. LI or sham surgery was introduced 14 days before ONC. Mice were euthanized 3 days after ONC. (**B**) Representative retinal sections showing RGCs stained for RBPMS and costained for p-S6, p-STAT3, or suppressor of cytokine signaling-3 (SOCS3). Scale bar, 50 μm. (**C**) Quantitation of normalized p-S6 immunostaining in RGCs shows increase with cLI compared with sham controls (unpaired *t* test, *P* = 0.0047, *n* = 5 mice per group). (**D**) Quantitation of normalized p-STAT3 immunostaining in RGCs shows increase with cLI compared with sham controls (unpaired *t* test, *P* = 0.0061, *n* = 5 mice in each group). (**E**) Quantitation of normalized SOCS3 intensity in RGCs shows small decrease with cLI compared with sham controls (unpaired *t* test, *P* = 0.0014; *n* = 5 mice per group). **P < 0.01. (**F**) Experimental timeline. DAPTA or PBS was injected i.p. daily beginning 2 days prior to LI and continuing through 1 day before ONC. LI or sham surgery was introduced 14 days before ONC. AMD3100 and P1 peptide were injected intraocularly (i.o.) immediately and 3 days after ONC. Mice were euthanized 14 days after ONC. (**G**) Representative longitudinal sections through the optic nerve showing similar levels of regenerating axons in mice receiving control treatments and growth factor inhibitors. White line, crush sites. Scale bar, 100 μm. (**H**) Quantitation of regenerating axons 1 mm and 1.5 mm from crush site in **G** (unpaired *t* test, *P* = 0.758; *n* = 3 or 4 mice per group).

Neuronal expression of phosphorylated signal transducer and activator of transcription 3 (p-STAT3), a marker of JAK/STAT3 pathway activation, or of phosphorylated ribosomal protein S6 (p-S6), a marker of mTOR pathway activation, is associated with the intrinsic regenerative ability of RGCs (30, 31). To assess STAT3 and mTOR activation in RGCs, we measured average fluorescence intensity of p-STAT3 and p-S6 as a semiquantitative reflection of intergroup differences. cLI mice showed a marked elevation of both p-S6 (~39%) and p-STAT3 (~47%) immunostaining in RNA-binding protein mRNA processing factor–positive (RBPMS^+^) RGCs 3 days after ONC compared with mice having sham surgery (Figure 4, A–D). Because deletion of SOCS3, a negative regulator of STAT3, in RGCs promotes optic nerve regeneration (32), we next investigated whether p-STAT3 elevation might be a consequence of SOCS3 downregulation. Following cLI, RGCs exhibited a small (~18%) but significant decrease of SOCS3 immunostaining compared with RGCs in sham controls (Figure 4, B and E). Downregulation of SOCS3 enables recombinant ciliary neurotrophic factor (CNTF) to elevate levels of p-STAT3 and to exert pro-regenerative effects in RGCs (32), raising the question of whether cLI sensitizes RGCs to CNTF. However, cLI did not enable CNTF to increase regeneration above the level induced by cLI alone (Supplemental Figure 4, D–F), arguing against a role for CNTF in the cLI effect.

Ocm and SDF1 are key mediators of zymosan-induced regeneration, while CCL5 mediates most of the pro-regenerative effects of virally mediated CNTF overexpression (10, 14, 16, 33). As positive controls, we verified that AMD3100, a selective antagonist of CXCR4, the primary receptor for SDF1, combined with P1 peptide, an Ocm antagonist, blocked approximately 70% of zymosan-induced regeneration (Supplemental Figure 4, A–C), while D-Ala-peptide T-amide (DAPTA), a selective antagonist to the CCL5 receptor CCR5, strongly suppressed regeneration induced by CNTF gene therapy (14). However, daily i.p. injection of DAPTA combined with intraocular injection of AMD3100 and P1 had no effect on regeneration induced by cLI (Figure 4, F–H).

Taken together, these results suggest that the activation of mTOR and STAT3 may be part of the downstream signaling pathway in cLI-induced regeneration because of mediators other than Ocm, SDF1, and CCL5, further emphasizing the difference between zymosan- and cLI-induced regeneration.

### Multiple LIs combined with PLX treatment enable brain reinnervation.

**Figure 5:**
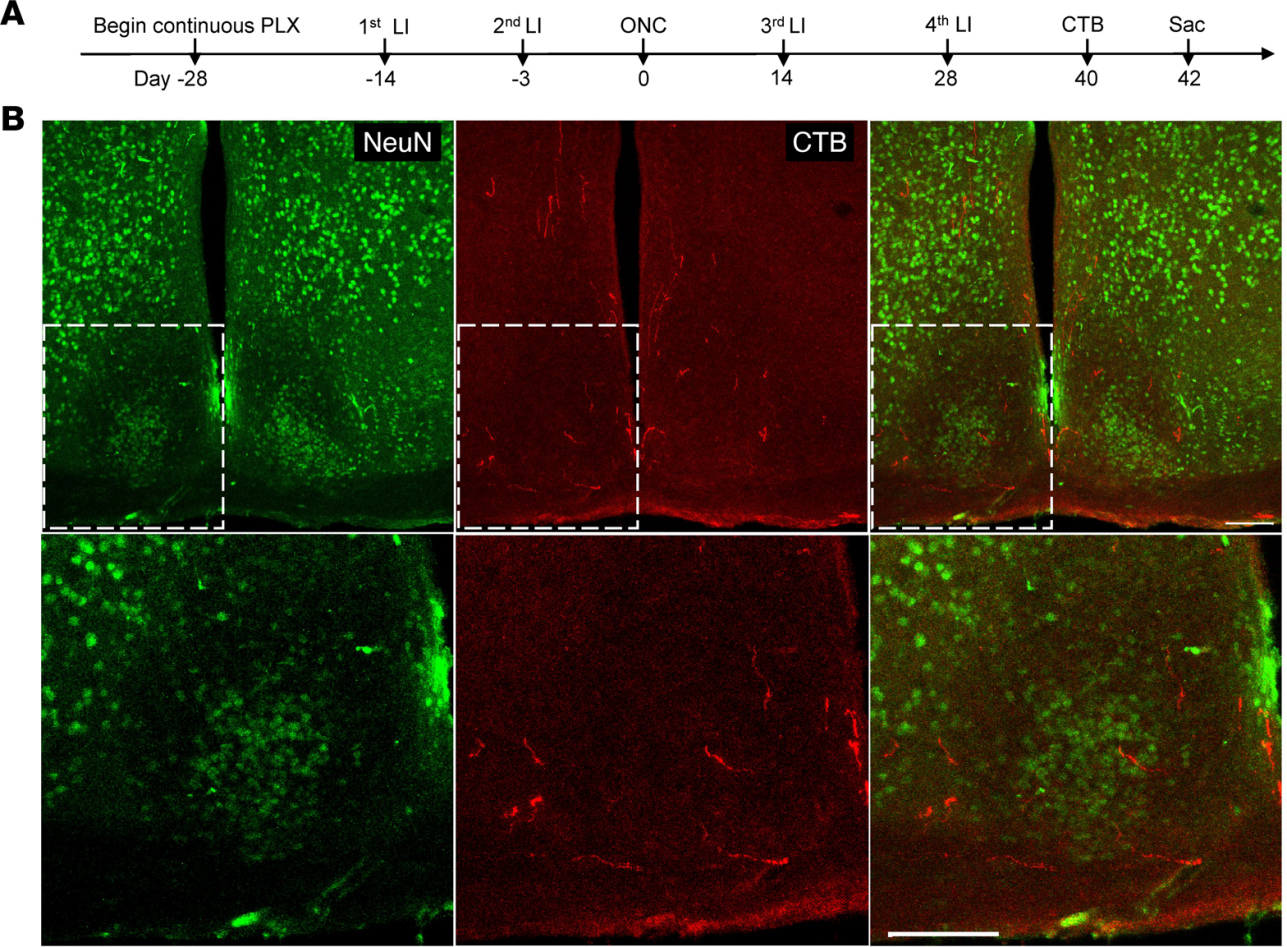
Multiple LIs combined with PLX enables brain reinnervation. (**A**) Experimental timeline. Mice received PLX treatment for 2 weeks before first LI until euthanasia. Multiple LIs were administered accordingly. Mice were euthanized 6 weeks after ONC. (**B**) Upper panels: brain sections stained with anti-NeuN and anti-CTB antibodies. Lower panels display magnified images of the areas in white dashed boxes in **A**, showing regenerating axons entering the core of the suprachiasmatic nucleus (SCN, visualized by NeuN immunostaining). Both scale bars, 100 μm.

As shown above, multiple episodes of LI induced full-length optic nerve regeneration but without brain reinnervation (Figure 1, F and G). PLX treatment combined with cLI resulted in more axons reaching the optic chiasm (Figure 2, B and C), raising the possibility that multiple episodes of LI combined with PLX (Figure 5A) might enable brain reinnervation. Six weeks after ONC, brain visual target areas including the suprachiasmatic nucleus (SCN), lateral geniculate nucleus (LGN), and superior colliculus (SC) were examined for regenerating axons. Three out of 4 PLX-treated mice showed regenerating axons in the SCN though not in the LGN or SC, with the best case showing some axons entering the SCN core (identified by NeuN immunostaining: Figure 5B). None of the mice fed control chow (*n* = 3) showed any brain innervation, as noted above. These findings demonstrate that brain reinnervation can be achieved by nongenetic manipulations.

## Discussion

**Figure 6:**
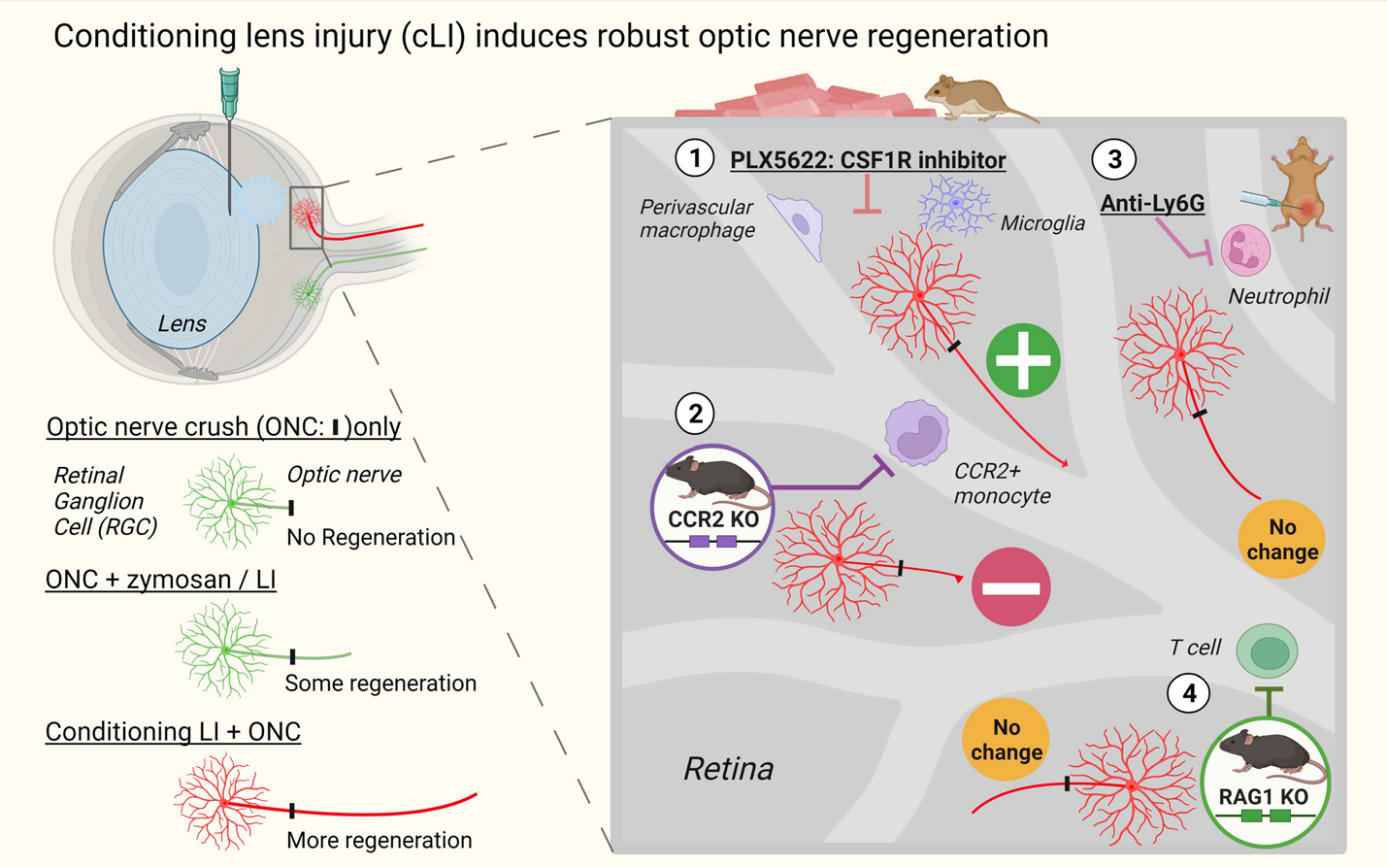
Schematics demonstrating robust optic nerve regeneration induced by cLI and whether perturbation of 4 major immune cell types affects cLI-induced regeneration using pharmacological or genetic manipulations.

Our results show that cLI promoted far stronger optic nerve regeneration than other inflammatory manipulations studied thus far that we know of (Figure 6). The effects of cLI greatly exceeded those of LI or zymosan post-ONC, whereas zymosan pre-ONC had almost no benefit at all. These findings, combined with the results of our loss-of-function studies, point to the existence of distinct molecular and cellular signals associated with cLI-induced regeneration.

Our lab previously identified neutrophil-derived Ocm and macrophage-derived SDF1 as key mediators of zymosan-induced regeneration (10, 11). Another widely used approach to induce optic nerve regeneration, CNTF gene therapy, also depends on neuroinflammation and elevation of yet another pro-regenerative factor, the chemokine CCL5 (14). Combining a blocking peptide for Ocm and the SDF1 antagonist AMD3100 suppresses the effects of zymosan, while the CCL5 antagonist DAPTA strongly suppresses the effects of CNTF gene therapy (14). However, a combination of all 3 inhibitors had no noticeable effect on regeneration induced by cLI. At the same time, whereas microglia were found to be irrelevant for regeneration induced by LI post-ONC (24), removal of resident macrophages using the same CSF1R inhibitor, PLX, strongly enhanced regeneration induced by cLI. This discrepancy may reflect a distinct local immune/metabolic landscape resulting from cLI combined with CSF1R inhibition prior to ONC, as seen in skeletal muscle repair (34). In addition, whereas depletion of mature neutrophils strongly suppresses optic nerve regeneration induced by zymosan (11) or CNTF gene therapy (14), multiple strategies to deplete mature neutrophils had no observable effect on regeneration induced by cLI. In several neurological diseases, T cells are implicated in beneficial outcome (25, 35), yet mature T cells were also found to be dispensable in our studies. The one manipulation that partially decreased regeneration was suppression of CCR2^+^ monocyte infiltration, suggesting that these cells do contribute, either directly or indirectly, to the effects of cLI on regeneration and that there may be a subset of pro-regenerative CCR2^+^ monocyte/macrophages related to those involved in the preconditioning peripheral nerve injury phenomenon (20). CCR2 deficiency preferentially affects the CCR2^+^ monocyte population, leaving open the possibility that a more comprehensive blockade of all monocytes could have a stronger effect (36).

A key challenge in optic nerve regeneration is to reconnect RGC axons with correct brain target areas to enable functional recovery (37). We show here that multiple, spaced episodes of LI enabled some RGCs to regenerate axons the full length of the optic nerve and that combining repeated LI with the CSF1R inhibitor PLX led to reinnervation of at least 1 central target area, the SCN, within 6 weeks. This level of regeneration is comparable to that of the most effective gene therapies described to date (38). RGCs fall into more than 40 subtypes based on functional, morphological, and molecular features, among which α-RGCs and melanopsin-positive intrinsically photosensitive RGCs preferentially show high levels of survival and regenerative capacity after deleting phosphatase and tensin homolog (Pten) (39). The additional deletion of SOCS3 while providing an adeno-associated virus expressing CNTF, which acts largely via CCL5, induces regeneration from a broad spectrum of RGCs (2), as does combining Pten deletion with zymosan and CPT-cAMP (16, 39, 40). In future studies, it will be important to investigate which RGC subtypes are stimulated by cLI, whether regenerating axons in the SCN form synapses to enable functional recovery, and whether the effects of cLI can be further augmented by counteracting other cell-autonomous and -nonautonomous suppressors of growth, e.g., inhibitory signals associated with myelin and the fibrotic scar (5, 41–43).

Several technical notes are in order. First, whereas an early study concluded that, to induce regeneration, LI needed to be severe and cataractogenic, reflecting considerable damage to the lens capsule, cortex, and nucleus (44), we found that mild injury to the lens posterior capsule and cortex that did not alter overall lens transparency was highly effective. Severe LI leads to shrinkage and rupture of the lens, making repeated episodes impossible. Repeated episodes of mild LI and optimal timing enabled us to push the potential of this manipulation to augment axon regeneration to exceptional levels. Second, regarding mouse genetic background, the strong phenotypes we observed in this study are from 129S1 mice. Different mouse strains show highly variable regenerative capacities in response to intraocular inflammation (28, 29), and comparative genetic studies could provide further insights into molecular bases of optic nerve regeneration. Third, it should be noted that the methods used here to manipulate major immune cell populations are likely to have incomplete and off-target effects, leaving the contribution of remnant and rare populations, e.g., immature neutrophils and innate lymphoid cells, to be further explored. Finally, whereas this study has focused on factors associated with inflammation, it remains possible that components of the lens per se contribute to regeneration or factors derived from other tissues as a result of cLI (45–47). Deeper unbiased analyses to identify changes in all neuronal and non-neuronal populations, cell type–specific transcriptional changes, and ligand-receptor interactions will be essential to better understand the mechanisms underlying the phenomenon described here (6, 7, 48).

In conclusion, cLI opens the possibility of identifying cell-nonautonomous targets to promote robust optic nerve regeneration without directly manipulating RGCs’ program of gene expression. With this potentially new paradigm in hand, we hope that cLI can be translated into potent therapies to address currently incurable neuronal and axonal losses after traumatic, ischemic, or degenerative damage to the CNS.

## Methods

### Mice.

WT 129S1 mice (129S1/SvImJ; strain 002448) and C57BL/6J mice (strain 000664) (6- to 12-week-old) of both sexes obtained from the Jackson Laboratory were used in this study. CCR2^RFP/RFP^ [B6.129(Cg)-CCR2tm2.1Ifc/J; strain 017586] and RAG1-KO (B6.129S7-*Rag1^tm1Mom^*/J; strain 002216) mice were originally purchased from the Jackson Laboratory. Homozygous CCR2^RFP/RFP^ mice were crossed with C57BL/6J mice to obtain heterozygous CCR2^RFP/+^ mice. Experimental CCR2^RFP/RFP^ mice and littermate CCR2^RFP/+^ controls were generated from heterozygous CCR2^RFP/+^ breeder pairs, maintained in C57BL/6J background, and genotyped by Transnetyx. All mice were housed under the same conditions for at least 4 days before being used in experiments and were maintained in regular cages on a 12-hour light/12-hour dark cycle with ad libitum access to regular food and water, except RAG1-KO mice and their matched control mice, which were maintained in autoclaved cages on a 12-hour light/12-hour dark cycle with ad libitum access to sterile food and water. All experiments used 6- to 12-week-old male and female 129S1 mice unless otherwise specified.

### Mild LI.

For surgery, mice were anesthetized by i.p. injection of ketamine and xylazine. Mild LI was performed by puncturing the lens posterior capsule and cortex to a depth of 0.5–1 mm using a disposable 30G sharp needle while sham surgery was performed by puncturing the posterior part of the eye into the vitreous chamber with care taken not to touch the lens. Mild LI results in a local response while maintaining overall transparency (Supplemental Figure 1A). All eyes with mild LI appear indistinguishable from those with sham surgery. Mice with severe damage to the whole lens, including capsule, cortex, and nucleus indicated by global or nucleus whiteness or disintegration of the lens, were excluded from the study.

### ONC and intravitreal injections.

For surgery, mice were anesthetized by i.p. injection of ketamine and xylazine. The optic nerve was intraorbitally crushed 0.5–1 mm behind the optic disc for 2–5 seconds using fine forceps (Dumont 5 FST), as described previously (10). Agents to be tested were injected intravitreally in a volume of 2 μL per eye using a 33G blunt needle to avoid any LI. Reagents injected intravitreally include zymosan (sterilized before use), CXCR4 antagonist AMD1000, P1 peptide, and CNTF recombinant protein. CTB or CTB-conjugated recombinant protein was injected intravitreally (2 μL per eye) to label regenerating axons 2 days before mice were euthanized. Details of regents are listed in Supplemental Table 1.

### Neutrophil depletion.

To deplete neutrophils systemically, mice received retro-orbital or i.p. injection of 100 μg anti-mouse Ly6G IgG antibody (BE0075-1; Bio X Cell) whereas control mice received isotype-matched IgG2a antibody (BE0089; Bio X Cell) using a modified protocol (49). To verify neutrophil depletion, blood neutrophils were evaluated by flow cytometry (LSRFortessa, BD Biosciences) using Mouse MDSC Flow Cocktail (147003; BioLegend) and analyzed using FlowJo V10 software (Tree Star).

### Resident macrophage depletion.

To deplete resident macrophages, mice were fed chow containing 1,200 parts per million PLX5622 (Plexxikon) ad libitum. Control mice received the same chow but without the drug. Chow consumption began 14 days prior to further experimental procedures and continued through the experiments to ensure sufficient and sustained depletion.

### Immunohistochemistry and imaging.

Mice were anesthetized and perfused through the heart with PBS followed by 4% paraformaldehyde (PFA). Eyes and optic nerves were dissected out. Eyes were carefully examined under a dissecting microscope (A60S Leica microscope) for cornea damage, intraocular bleeding, severe lens damage, or eye dystrophy, which were used as criteria for exclusion. Tissues were then postfixed for 1 hour in 4% PFA, transferred to 30% sucrose overnight (4°C), and embedded in Tissue-Tek (Sakura). Frozen sections (14 μm) were cut longitudinally on a cryostat, thaw-mounted onto glass slides (Superfrost plus, Thermo Fisher Scientific), and stored at –80°C until further use. Antibodies are listed in Supplemental Table 1. Images were taken using a Nikon E800 or Zeiss LSM710 confocal microscope, then merged, cropped, and optimized using ImageJ (Fiji).

### Quantitation of regenerating axons in the optic nerve.

Axon regeneration was quantified as described previously (8). In brief, the number of CTB-positive axons extending prespecified distances from the injury site were counted under 400× original magnification in 3–4 sections per sample. These values were normalized to the cross-sectional area of the optic nerve and extrapolated to the whole optic nerve.

### Quantitation of RGCs in whole-mounted retina.

For quantitation of surviving RGCs/mm^2^, retinal flatmounts were stained with an antibody against βIII-tubulin. Retinas were divided into 4 quadrants. In each quadrant 2 independent fields were sampled, representing the center and periphery. The average number of βIII-tubulin–positive RGCs per field was determined and divided by the area of the field. Values were averaged per retina. At least 4 retinas from no fewer than 3 mice per condition were analyzed.

### Preparation and staining of whole eye sections.

After perfusing mice as described above, eyes were collected and postfixed in 4% PFA for 1 hour, transferred to 30% sucrose overnight at 4°C, and frozen sectioned at 14 μm. Sections were incubated with primary antibodies at 4°C overnight after blocking with appropriate sera for 1 hour at room temperature. After washing 3 times, sections were incubated with the appropriate fluorescent secondary antibody and DAPI and then mounted. Primary antibodies used in this study are listed in Supplemental Table 1.

### Preparation and staining of brain sections.

Mice were perfused as described above. Brains were postfixed for 48 hours at 4°C, then transferred to 30% sucrose until they sank, embedded in O.C.T., frozen, and cryostat-sectioned in the coronal plane at 50 μm. Sections were collected and stained free-floating in PBS to visualize CTB-labeled growing axons and NeuN (to visualize brain structures), then mounted onto slides.

### Analysis of p-S6, p-STAT3, and SOCS3 levels in RGCs.

To analyze p-S6, p-STAT3, and SOCS3 level in RGCs, 4 nonadjacent retinal sections from each mouse were stained simultaneously with anti-RBPMS antibodies following the steps mentioned above (see *Preparation and staining of whole eye sections*). To measure fluorescence intensity of p-S6, p-STAT3, and SOCS3 immunostaining in RGCs, images were acquired simultaneously with identical configurations and then analyzed for each retina. At least 200 RGCs per eye in at least 3 mice per group were manually selected and measured for mean fluorescence intensity per cell using ImageJ.

### Statistics.

Statistical analyses were done with GraphPad Prism 8, and the significance level was set at *P* < 0.05. For comparisons between 2 groups, 2-tailed unpaired or paired *t* tests were used. One-way ANOVA followed by Tukey’s multiple-comparison test were performed for comparisons among 3 or more groups. Data are represented as mean ± SEM; *P* values of post hoc analyses are reported in the figures. All details regarding statistical analyses, including the tests used, *P* values, exact values of *n*, and definitions of *n*, are described in the figure legends.

### Study approval.

Experiments were performed at Boston Children’s Hospital with approval from the Institutional Animal Care and Use Committee.

## Author contributions

QF was responsible for conceptualization, investigation, methodology, formal analysis, and drafting, reviewing, and editing of the manuscript; KAW was responsible for investigation, methodology, and resources; and LIB was responsible for conceptualization, methodology, supervision, funding acquisition, and reviewing and editing of the manuscript.

## Supplementary Material

Supplemental data

## Version 1 02/23/2023

In-Press Preview

## Version 2 04/10/2023

Electronic publication
