# Supplementary material for: Full-length optic nerve regeneration in the absence of genetic manipulations
Source: JCI Insight. 2023 Apr 10;8(7):e164579. doi: 10.1172/jci.insight.164579 (PMC10132151; doi:10.1172/jci.insight.164579)
Supplement: Supplemental data [file jciinsight-8-164579-s125.pdf]

Figure S1

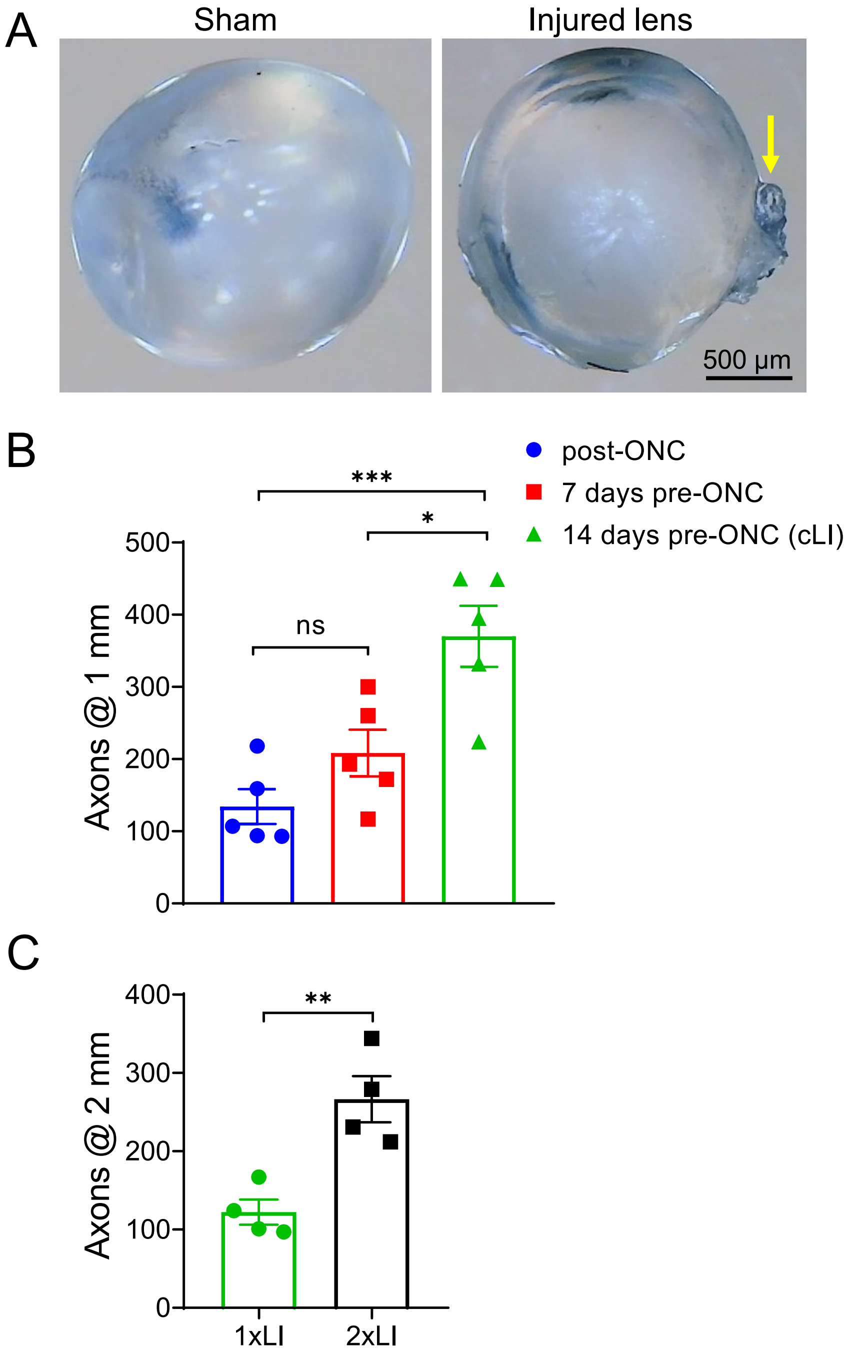

A. Representative lens images 14 days after sham surgery or mild lens injury showing local “volcano-like” structure on the surface of the injured lens indicated by the yellow arrow while maintaining overall transparency. Scale bar, 500 μm.

B. Quantitation of regenerating axons 1 mm from crush site when LI was applied immediately after ONC (post-ONC), 7 days before ONC (7 days pre-ONC) and 14 days before ONC (14 days pre-ONC: cLI) (One-way ANOVA followed by Tukey’s multiple comparisons test; post-ONC vs. 7 days pre-ONC $p = 0.3034$; post-ONC vs. 14 days pre-ONC $p = 0.0009$; 7 days pre-ONC vs. 14 days pre-ONC $p=0.0141$; $n = 3$ or 4 mice per group).

C. Quantitation of regenerating axons 2 mm from crush site after one (1xLI) or two LI (2x LI) (unpaired t-test, $p = 0.0051$; $n = 3$ mice per group).

Figure S2

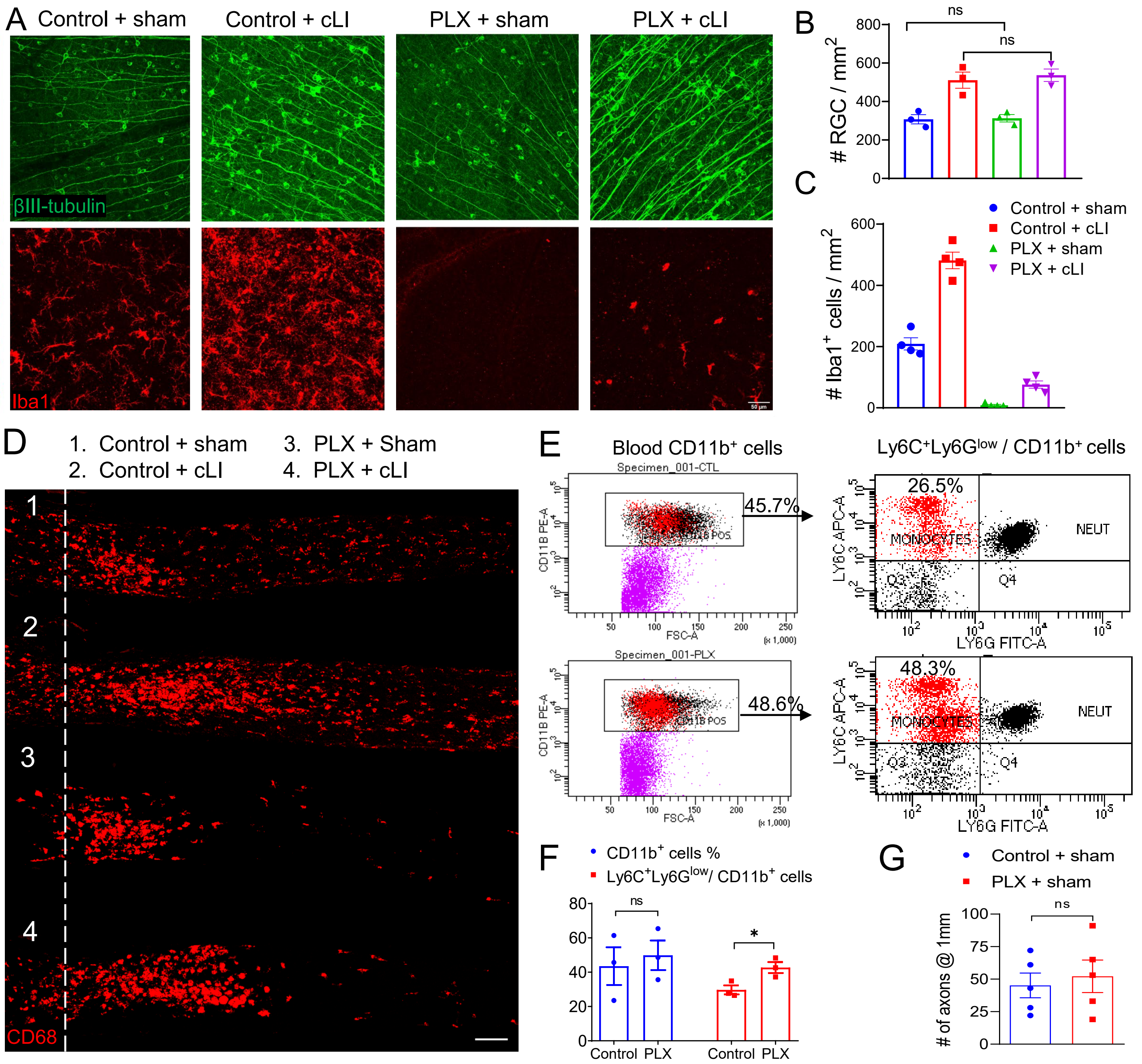

A. Representative whole-mounted retinas showing βIII-tubulin labeled RGCs and Iba1 labeled macrophages 4 weeks after ONC. Scale bar, 50 μm.

B. Quantitation of surviving βIII-tubulin+ RGCs in (A) (one-way ANOVA followed by Tukey's multiple comparisons test; Control + Sham vs. PLX + Sham, p= 0.9993; Control + cLI vs. PLX + cLI, p= 0.9316; n=3 mice per group).

C. Quantitation of Iba1⁺ cells in (A) (Mean numbers are 209 in Control + sham vs. 481 in Control + cLI vs. 5 in PLX + sham vs. 66 in PLX + cLI).

D. Representative longitudinal sections through the optic nerve from Fig 2 co-labeled for CD68. A population of CD68⁺ cells persisted in the optic nerve especially around crush sites after PLX treatment combined with sham surgery or cLI. *White line*: crush site. Scale bar, 100 μm.

E. Flow cytometric analysis of blood collected from mice receiving PLX chow or control chow after 4 weeks. Dot-plot of monocytes (CD11b⁺ Ly6C⁺Ly6Glow) in blood. The percentages of CD11b⁺ cells and monocytes in CD11b⁺ cells are shown.

F. Quantitation of the percentages of CD11b⁺ cells and monocytes in CD11b⁺ cells in (D) (unpaired t-test; CD11b⁺ cell %: control vs. PLX, p = 0.6739; monocytes / CD11b⁺ cells: control vs. PLX, p = 0.0349; n = 3 mice per group).

G. Quantitation of regenerating axons 1 mm from crush site in *Fig 2B.1-2* (unpaired t-test, p = 0.668, n = 5 mice per group).

Figure S3

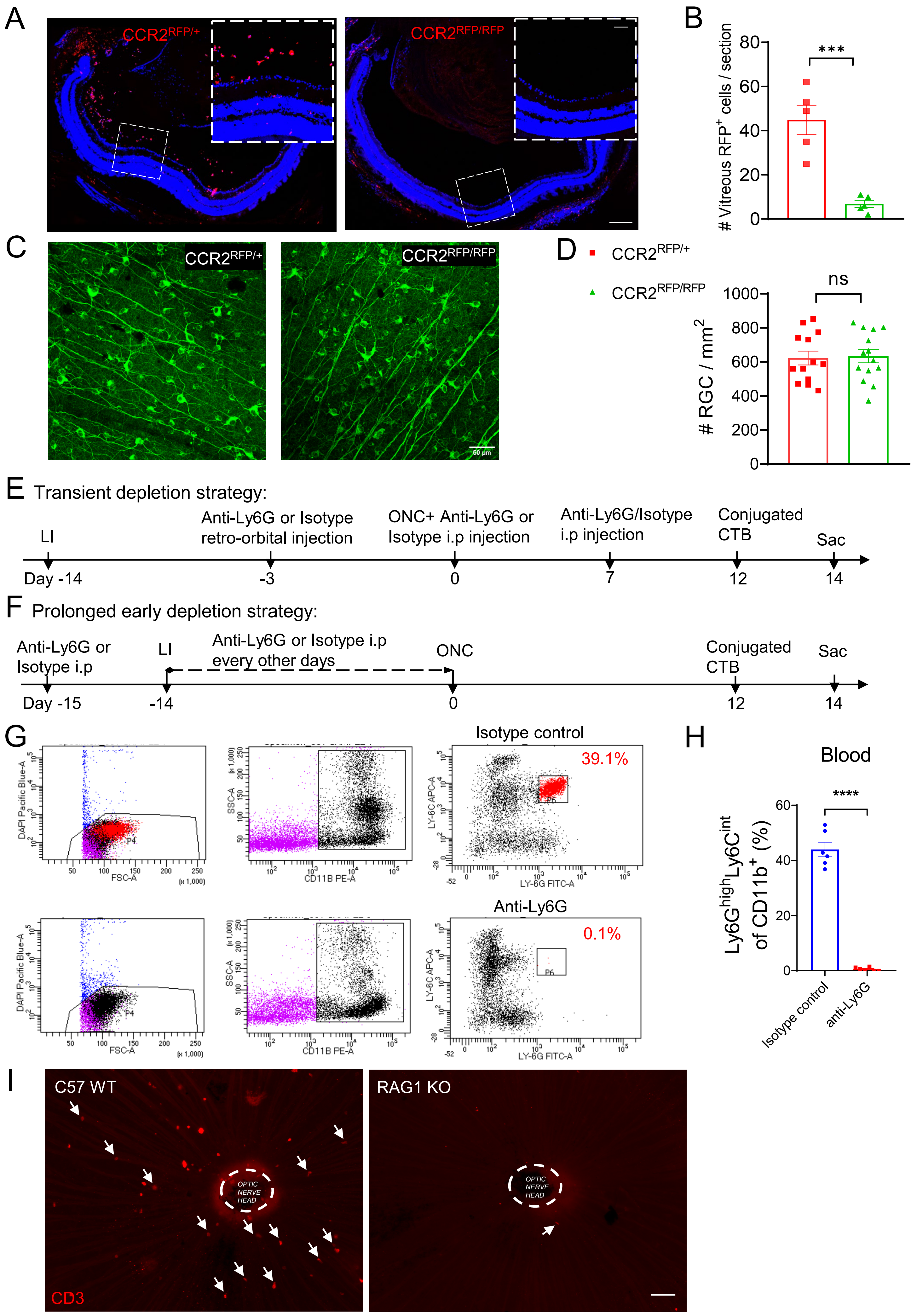

A
CCR2^RFP/+
CCR2^RFP/RFP
B
Vitreous RFP+ cells / section
***
0
20
40
60
80
C
CCR2^RFP/+
CCR2^RFP/RFP
50 µm
D
CCR2^RFP/+
CCR2^RFP/RFP
ns
RGC / mm2
0
200
400
600
800
1000
E Transient depletion strategy:
LI
Anti-Ly6G or Isotype retro-orbital injection
ONC+ Anti-Ly6G or Isotype i.p injection
Anti-Ly6G/Isotype i.p injection
Conjugated CTB
Sac
Day -14
-3
0
7
12
14
F Prolonged early depletion strategy:
Anti-Ly6G or Isotype i.p
LI
Anti-Ly6G or Isotype i.p every other days
ONC
Conjugated CTB
Sac
Day -15
-14
0
12
14
G
Isotype control
39.1%
Anti-Ly6G
0.1%
DAPI Pacific Blue-A
FSC-A
SSC-A
CD11B PE-A
LY-6C APC-A
LY-6G FITC-A
P4
P6
H
Blood
****
Ly6G^high Ly6C^int of CD11b+ (%)
0
20
40
60
Isotype control
anti-Ly6G
I
C57 WT
RAG1 KO
OPTIC NERVE HEAD
CD3

Figure S3

A. Representative whole eye sections showing many fewer RFP^+ cells in the vitreous chamber in $CCR2^{RFP/RFP}$ mice than in $CCR2^{RFP/+}$ mice. The white dashed boxes are the magnified images showing RFP^+ cells. Scale bar, 200 μm; Enlarged scale bar, 50 μm.

B. Quantitation of vitreous RFP^+ cell number in (A) (unpaired t-test, $p = 0.0005$; $n = 5$ mice in each group; 4 non-sequential sections were analyzed for each mouse).

C. Representative whole-mounted retinas showing βIII-tubulin labeled RGCs in $CCR2^{RFP/RFP}$ mice and $CCR2^{RFP/+}$ mice. Scale bar, 50 μm.

D. Quantitation of βIII-tubulin$^+$ RGCs in (C) (unpaired t-test, $p= 0.853$; $n = 6$ or 7 mice in each group).

E. Experimental timeline for transient depletion strategy. LI was introduced in 129S WT mice 14 days before ONC. Mice were euthanized 14 days after ONC.

F. Experimental timeline for prolonged early depletion strategy. LI was introduced in 129S WT mice 14 days before ONC. Mice were euthanized 14 days after ONC.

G. Flow cytometric analysis of blood collected from mice receiving isotype control or anti-Ly6G injections. Dotplot of mature neutrophils ($CD11b^+$ $Ly6G^{high}Ly6C^{int}$) in blood 14 days post-injections using prolonged late depletion strategy (Fig. 3D). The percentages of neutrophils in $CD11b^+$ cells are shown.

H. Quantitation of percentage of mature neutrophils in $CD11b^+$ cells in (G) (unpaired t-test, $p < 0.0001$; $n = 6$ mice per group).

I. Representative whole-mounted retinas showing near-absence of $CD3^+$ cells near the optic nerve head in RAG1 KO mice compared to C57 WT mice. *White arrow*: $CD3^+$ cell. Scale bar, 50 μm.

Figure S4

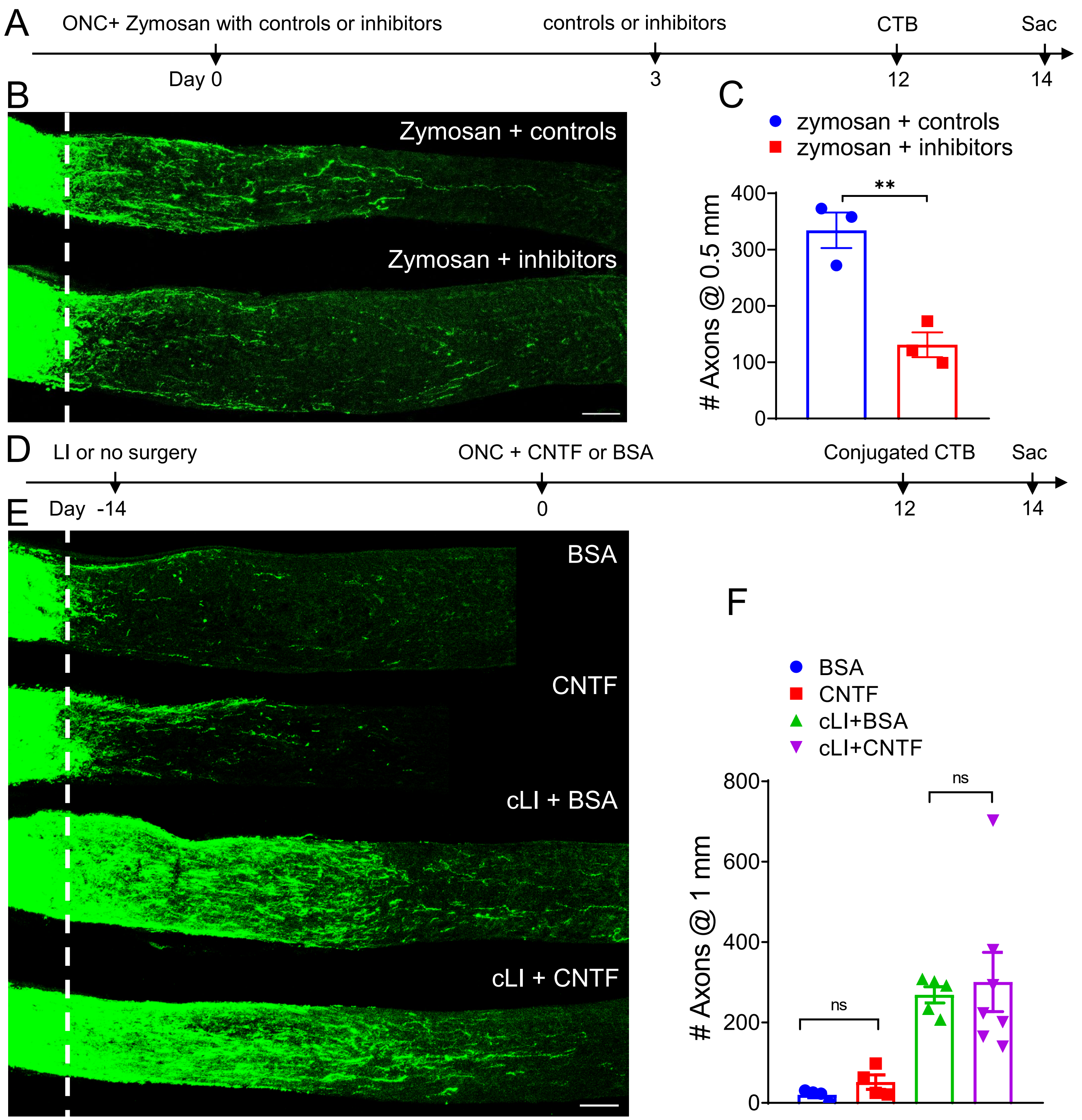

A. Experimental timeline. Zymosan with control peptide or with inhibitors (AMD3100 and P1 peptide) were injected intraocularly (*i.o.*) immediately after and 3 days after ONC. Mice were euthanized 14 days later.

B. Representative longitudinal sections through the optic nerve showing many fewer regenerating axons in mice receiving zymosan plus inhibitors than in mice receiving zymosan plus controls. *White line:* crush site. Scale bar, 100 μm.

C. Quantitation of regenerating axons at 0.5 mm from crush sites in (C) (unpaired t-test, 0.0061; n = 3 mice per group).

D. Experimental timeline. LI (or no surgery) was introduced 14 days before ONC, and CNTF or BSA were injected intraocularly (i.o) immediately after ONC. Mice were euthanized 14 days later.

E. Longitudinal sections through the optic nerve showing regenerating axons 14 days post-ONC. CNTF injection alone post-ONC resulted in few regenerating axons, and cLI combined with CNTF (cLI + CNTF) showed similar levels of regeneration as cLI combined with BSA (cLI + BSA). *White line* indicates crush sites. Scale bar, 100 μm.

F. Quantitation of regenerating axons 1 mm from crush sites in (C) (unpaired t-test, BSA vs. CNTF, p = 0.152; cLI + BSA vs. cLI + CNTF, p = 0.73; n = 4 or 5 mice each group).

Key Resources Table

Reagent type (species) or resource	Designation	Source or reference	Identifiers	Additional information
Strain, strain background (*Mus musculus, male and female*)	129S1/SvImJ	The Jackson Laboratories	002448	
Strain, strain background (*Mus musculus, male and female*)	C57BL/6J	The Jackson Laboratories	000664	
Genetic strain, strain background (*Mus musculcus, male and female*)	B6.129(Cg)-CCR2tm2.1Ifc/J	The Jackson Laboratories	017586	
Genetic strain, strain background (*Mus musculcus, male and female*)	B6.129S7-Rag1tm1Mom/J	The Jackson Laboratories	002216	
Antibody	Rabbit anti-Cholera toxin subunit B (CTB)	GenWay Biotech	GWB-7B96E4	IF (1:500)
Antibody	Rabbit polyclonal anti-beta III Tubulin	Abcam	ab18207	IF (1:500)
Antibody	Rabbit polyclonal anti-Iba1	WAKO	Cat: #019-19741	IF (1:1000)

Antibody	Rat monoclonal anti-mouse CD68	Bio-Rad	MCA1957	IF (1:500)
Antibody	Anti-NeuN, Clone: A60	Fisher Scientific	Mab377	IF (1:200)
Antibody	Phospho-S6 Ribosomal Protein (Ser240/244) Rabbit mAb	Cell Signaling Technology	5364	IF (1:200)
Antibody	Guinea Pig Polyclonal anti-RBPMS	PhosphoSolutions	Cat# 1832-RBPMS	IF (1:500)
Antibody	Phospho-Stat3 (Tyr705) Rabbit monoclonal Ab	Cell Signaling Technology	Cat# 9145	IF (1:200)
Antibody	Mouse SOCS3 Monoclonal Antibody	Fisher Scientific	MA1-19373	IF (1:500)
Antibody	RFP Antibody Rabbit Polyclonal	Rockland	600-401-379	IF (1:500)
Antibody	Anti-mouse Ly6G	Bio X Cell	BP0075	200 µg in 200 µl (i.p.)
Antibody	Rat IgG2a isotype control	Bio X Cell	BE0089	200 µg in 200 µl (i.p.)
Antibody	Mouse CD3 Antibody	R&D Systems	MAB4841	IF (1:100)
Antibody	Rat monoclonal anti-mouse CD16/32 (mouse Fc block)	BioLegend	156603	FACS
Antibody	Mouse/rabbit/guinea pig/goat IgG conjugated to Alexa Fluor 488/555/594	Thermo Fisher Scientific	https://www.thermofisher.com/antibody/secondary/query/alexa	1:500

Commercial assay or kit	Mouse MDSC Flow Cocktail 2 with Isotype Ctrl	BioLegend	147003	FACS
Commercial assay or kit	OCT compound	Sakura Finetek	4583	
Peptide/recombinant protein	CNTF	Pepro Tech	AF-450-50	0.5 µg/µl
Peptide/recombinant protein	CTB	Sigma	SAE0069	2 µg/µl
Peptide/recombinant protein	CTB-Alexa Fluor 555/488 Conjugate	Thermo Fisher Scientific	C-22843 /C-22841	2 µg/µl
Peptide/recombinant protein	P1	Genscript Corp (ref. 28)	synthesized	2.3 µg/µL
Peptide/recombinant protein	Bovine Serum Albumin (BSA)	Sigma-Aldrich	A9085	0.5 µg/µl
Chemical compound	DAPTA	Selleck Chemicals	S8501	10 µg/kg
Chemical compound	PLX5622	Plexxikon Inc.		1200 ppm in chow
Chemical compound	RBC Lysis Buffer (10X)	BioLegend	420301	
Chemical compound	AMD3100	Sigma	CAS155148-31-5	100 µM
Chemical compound	zymosan	Sigma	Z4250	12.5 µg/µL
Software, algorithm	Zen	Zeiss		
Software, algorithm	Fiji (ImageJ)			

Software, algorithm	GraphPad Prism	GraphPad	Version 8	
Software, algorithm	BioRender	BioRender		
Other	DAPI	Sigma-Aldrich	Cat: #9542	1:10000
